# Generation and characterisation of *Porphyromonas gingivalis* mutant lacking peptidylarginine deiminase activity

**DOI:** 10.1080/20002297.2017.1325258

**Published:** 2017-06-09

**Authors:** Estefanía Muñoz-Atienza, Joseph Aduse-Opoku, Magdalena B. Flak, Nikolay A. Paramonov, Costantino Pitzalis, Michael A. Curtis

**Affiliations:** ^a^ Institute of Dentistry, Barts and The London School of Medicine & Dentistry, Queen Mary University of London, London, UK; ^b^ Centre for Experimental Medicine & Rheumatology, William Harvey Research Institute, Barts and The London School of Medicine & Dentistry, Queen Mary University of London, London, UK

## Abstract

*Porphyromonas gingivalis* peptidylarginine deiminase (PPAD) is the focus of several studies due to its ability to citrullinate *in vitro* human proteins, which have been linked to the aetiopathogenesis of rheumatoid arthritis (RA). The aim of this work was the generation by homologous recombination and characterisation of a *P. gingivalis* W50 mutant lacking *pad* gene (PG1424) to study the role of PPAD in RA. To confirm the absence of PPAD activity in *P. gingivalis* PG1424, cells were incubated with arginine-containing substrates and citrullination of L-arginine measured using a colorimetric assay and thin-layer chromatography. Furthermore, arginine and lysine protease (gingipain) activities were assessed and immunoblotting was performed using monoclonal antibody 1B5 (mAb1B5) and a commercial anti-modified citrulline antibody (AMC) to detect differences in virulence factor expression. The deletion of *pad* gene in *P. gingivalis* PG1424 completely abolished the ability to autocitrullinate *P. gingivalis* proteins in the mutant strain and also the citrullination of used substrates but not free L-arginine. Moreover, the wild-type and mutant strains had similar total gingipain activities and reactivity with mAb1B5. In conclusion, this work has produced a well-characterised PPAD-deleted *P. gingivalis* strain, which can be used to help determine the role of citrullination by this microorganism in RA.

